# Is Nrf2 Behind Endogenous Neuroprotection of the Hippocampal CA2-4,DG Region?

**DOI:** 10.1007/s12035-022-03166-x

**Published:** 2022-12-22

**Authors:** Anita Lewczuk, Barbara Zablocka, Malgorzata Beresewicz-Haller

**Affiliations:** Mossakowski Medical Research Institute, Polish Academy of Sciences, Warsaw, Poland

**Keywords:** Endogenous neuroprotection, Cerebral ischemia, Nrf2, Sub-regional differences in the hippocampus

## Abstract

The transcription factor nuclear factor-erythroid 2-related factor 2 (Nrf2) is the master regulator of genes known to be involved in antioxidant, and anti-inflammatory processes, metabolic regulation, and other cellular functions. Here, we also hypothesize a core role for it in endogenous neuroprotection, i.e., the natural adaptive mechanisms protecting the brain from ischemia–reperfusion (I/R) episode. An example of endogenous neuroprotection is ischemia-resistance of the hippocampal regions comprising the CA2, CA3, CA4 and dentate gyrus subfields (here abbreviated to CA2-4,DG) which can be contrasted with the ischemia-vulnerable CA1 region. In the work detailed here, we used a gerbil model of transient cerebral ischemia to examined Nrf2 activation in CA1 and CA2-4,DG, in a control group, and post I/R episode. Data obtained indicate enhanced Nrf2 activity in CA2-4,DG as compared with CA1 in the control, with this difference seen to persist even after I/R. While I/R does indeed cause further activation of Nrf2 in CA2-4,DG, it is associated with slight and transient activation in CA1. Sub-regional differences in Nrf2 activity correlate with immunoreactivity of Keap1 (an Nrf2 suppressor) and Nrf2 target proteins, including heme oxygenase 1, the catalytic and modulatory sub-units of glutamate-cysteine ligase, and glutathione peroxidase 1. Pharmacological Nrf2 activation by sulforaphane results in protection of CA1 after I/R episode. Our results therefore suggest that high Nrf2 activity in CA2-4,DG may guarantee resistance of this region to I/R, potentially explaining the differential sensitivities of the hippocampal regions.

## Introduction

Ischemic stroke is a leading cause of death and long-term disability worldwide. And while significant progress has been made in understanding the pathophysiology of stroke over the past few decades, this has unfortunately not translated into progress in developing effective therapies [1]. So the search for effective neuroprotection goes on.

To date, attempts to identify neuroprotective targets have involved the study of ischemia-induced molecular cascades and the development of ways of suppressing them. The cascades in question entail disrupted oxidative phosphorylation that results in energy depletion and ion imbalance, as followed by cell-membrane depolarization, calcium overload, and extracellular accumulation of the excitatory amino acid glutamate. In turn, excitotoxic stress leads to cell death, accompanied by the formation of free radicals, swelling, and inflammation [2, 3]. While these consequences are known, to date, no compelling data have been published regarding efficacy of any pharmacologic or other therapies. This state of affairs implies a need for new approaches to the search for effective neuroprotection [4, 5]. So it is that the activation of natural adaptive mechanisms (called endogenous neuroprotection) is now discussed increasingly as a promising therapeutic method [5]. The aim of the approach here is to enhance and stimulate endogenous processes of plasticity and protection of the neuronal system that trigger the brain’s intrinsic capacity for self-defense.

The classic example of endogenous neuroprotection is “ischemic preconditioning” (a process whereby a short-duration, non-damaging ischemia–reperfusion episode evokes tissue resistance to consecutive long-lasting damaging ischemia) [6]. Another example is ischemia-resistance of the hippocampal regions comprising the CA2, CA3, CA4 and dentate gyrus subfields (here abbreviated to CA2-4,DG) as opposed to the ischemia-vulnerable CA1 region, which is to be noted in rodents as well as human beings [7–12]. This may be a phenomenon of scientific and translational significance.

The work detailed here has seen us investigate the role of the transcription factor known as Nuclear factor-erythroid 2-related factor 2 (Nrf2) in the endogenous neuroprotection of CA2-4,DG following ishemia-reperfusion (I/R) episode. The rationale for our research into Nrf2 has been twofold, reflecting (i) the status of Nrf2 as the master regulator of antioxidant defense and cellular stress resistance [13, 14] and (ii) the contribution of Nrf2 to cellular homeostasis that has become evident through many studies using cell lines and animal models, with consequent major raising of attention when it comes to the targeting of clinical potential [15–26].

**Fig. 1:**
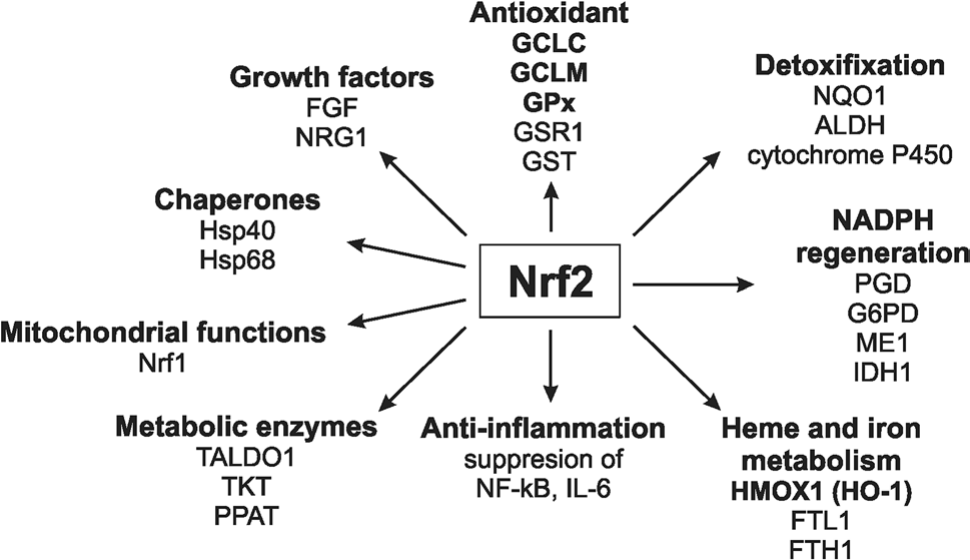
Graphical overview of selected Nrf2 target genes grouped by its function. Nrf2 is best known for its contribution to cellular defense mechanisms against oxidative stress. It coordinates antioxidant, detoxification, and NADPH-regeneration enzymes. Among antioxidants Nrf2 controls range of enzymes involved in glutathione homeostasis by regulating the expression of the catalytic (GCLC) and modulatory (GCLM) sub-units of glutamate-cysteine ligase (which catalyze glutathione biosynthesis), glutathione peroxidase (GPx), glutathione reductase (GSR1), and several glutathione S-transferases (GSTs). Moreover, Nrf2 regulates four enzymes involved in the generation of NADPH necessary for glutathione reduction, i.e., phosphogluconate dehydrogenase (PGD), glucose-6-phosphate dehydrogenase (G6PD), malic enzyme 1 (ME1), and isocitrate dehydrogenase 1 (IDH1). Additionally, Nrf2 controls the expression of a range of detoxification enzymes including NAD(P)H quinone dehydrogenase (NQO1), aldehyde dehydrogenases (ALDH), and cytochrome P450. Nrf2 also affects heme and iron metabolism with heme oxygenase-1 (HO-1) as the best known representative. It also plays a role in anti-inflammation throuhg inhibition of NF-κB and expression of its target genes. Moreover, Nrf2 controls growth factors, chaperones, and genes involved in metabolic processes and mitochondrial functions, and many more. Protein levels of genes highlighted in bold were measured in this study. Other abbreviations: FTL1-ferritin light chain 1, FTH1-ferritin heavy chain 1, TALDO1-transaldolase 1, TKT-transketolase, NGR1-neuregulin 1, PPAT-phosphoribosyl pyrophosphate amidotransferase

Nrf2 is expressed ubiquitously in various cell types, including neurons [27]. Under basal conditions, Nrf2 is sequestered in the cytoplasm through association with its repressor protein Keap1 (Kelch-like ECH-associated protein 1), which promotes Nrf2 polyubiquitination and subsequent proteasomal degradation [28]. However, in the presence of oxidative stress or inducers (often electrophiles), Nrf2 is freed from its repressor and is translocated directly into the nucleus. It binds to antioxidant response elements (ARE) in the promoter region of its target genes and promotes their transcription [29]. Currently, the list of genes possessing ARE runs to several hundred items, including genes encoding antioxidant and anti-inflammatory enzymes, heme, and iron metabolism, proteins that regulate the expression of other transcription factors and growth factors, genes involved in metabolic processes, and many more [13, 30, 31]. Classic examples of the genes are provided by heme oxygenase 1 (HO-1), the catalytic (GCLC), and modulatory (GCLM) sub-units of glutamate-cysteine ligase, and glutathione peroxidase 1 (GPx1), which are all shown together along with others in Fig. 1.

Numerous lines of evidence demonstrate the beneficial contribution of Nrf2 to protection against oxidative damage in various experimental models, including of cerebral ischemia [22, 32, 33]. Nrf2 deletion has been shown to render animals significantly more sensitive to the damaging effects of ischemia and reperfusion injury than are their wild-type counterparts [34]. Conversely, in various models of cerebral ischemia pharmacological Nrf2 activation is seen to exert protective effects [15, 18, 22, 35, 36].

Of the many different Nrf2 activators, the one receiving most attention is sulforaphane (SFN) [37–40] — an isothiocyanate obtained from cruciferous vegetables such as broccoli. This has high bioavailability due to its low molecular weight and marked capacity to cross the blood–brain barrier (BBB) [20]. There are many reports indicating a beneficial role for SFN in protecting against cerebral ischemia, as well as various neurodegenerative disorders [15–18, 20–22, 35, 36].

We drew on literature data to hypothesize that Nrf2 may be responsible for the endogenous resistance of hippocampal CA2-4,DG. To verify this, we used a gerbil model of transient cerebral ischemia to examine Nrf2 activity in CA1 and CA2-4,DG in control gerbils, and in animals studied different lengths of time after I/R episode. The analysis indicates that CA2-4,DG has higher Nrf2 activity than CA1 in control hippocampi, with this difference persisting even after I/R, which is seen to exert differential effects on the two hippocampal regions. Ischemia and reperfusion injury cause further activation of Nrf2 in CA2-4,DG; as well as a slight, transient activation in CA1.

The observed relationship in Nrf2 activity differentiating the two hippocampal regions correlates with immunoreactivity due to Keap1, as well as with the presence of Nrf2 target proteins such as HO-1, GLCL, GCLM and GPx1. Additionally, pharmacological activation of Nrf2 is found to protect neurons in the hippocampal CA1 region following I/R episode. These results suggest that high levels of nuclear Nrf2 activity in CA2-4,DG may guarantee resistance of this region to I/R episode, at the same time offering an explanation for the phenomenon whereby the hippocampal regions are of differing sensitivity.

## Materials and Methods

### Ethical Statement and Animals

All experimental procedures were approved by the Local Ethics Committee for Animal Experimentation, and efforts were made to minimize animal suffering. The experimental Wistar rats (6-7-day-old pups) and Mongolian gerbils (*Meriones unguiculatus*) were obtained from the Animal House of the Mossakowski Medical Research Institute of the Polish Academy of Sciences.

### Transient Cerebral Ischemia in Gerbils

Gerbils weighing 60–70 g were made subject to transient cerebral ischemia by way of 5-min bilateral ligation of the common carotid arteries under isoflurane anesthesia, and the closely-controlled normothermic conditions described previously [41, 42]. Following ischemia, animals recovered for 15 and 30 min or 1, 2, 24, 36, 48, and 72 h, prior to CA1 and CA2-4,DG regions of the hippocampus being isolated to obtain nuclear and cytoplasmic fractions for western blot analysis. Hippocampi from untreated animals were used as controls. In addition, whole brains were isolated for histopathological purposes 7 days after ischemia and/or sulforaphane treatment. Sulforaphane, a commonly used Nrf2 activator, was applied dissolved in saline and injected intraperitoneally at a dose of 5 mg/kg during the course of the ischemic insult (I/R SFN) or 30 min of reperfusion (I/R 30’SFN) [15, 38]. The dose used was selected by reference to literature analysis claiming effectiveness in many models describing diseases of the nervous system [31].

### Isolation of Nuclear and Cytoplasmic Fractions

Nuclear and cytoplasmic fractions were obtained using a nuclear extraction kit, in accordance with the instructions of the manufacturer (Cayman Chemical). Isolated CA1 and CA2-4,DG regions of the hippocampus were subject to brief homogenization in ice-cold complete hypotonic buffer, as supplemented with DTT and NP-40. Then, after 15 min of incubation, they were centrifuged at 300 × *g* for 10 min at 4°C. The pellet was re-homogenized in complete hypotonic buffer, incubated for an additional 15 min, supplemented with 10% NP-40, and centrifuged at 14,000 × *g* for 30 s at 4°C. The supernatants obtained from two successive centrifugations were combined and processed as the cytoplasmic fractions. The pellet was resuspended in ice-cold complete nuclear extraction buffer, incubated for 15 min, and centrifuged at 14,000 × *g* for 10 min at 4°C to obtain the supernatant containing nuclear fractions. The purity of the nuclear and cytoplasmic fractions was confirmed by western blotting, with respective use made of anti-THOC and anti-LDHB antibodies as nuclear and cytoplasmic markers (data not shown).

### Western Blot Analysis

**Fig. 2:**
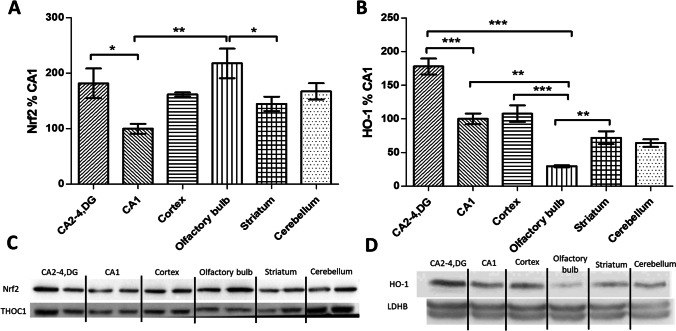
Nrf2 activity and heme oxygenase 1 (HO-1) immunoreactivity in various brain areas in control gerbils: CA2-4,DG and CA1 (as ischemia-resistant and vulnerable hippocampal regions), cortex, olfactory bulb, striatum, and cerebellum. **A** Nuclear (30 µg) or **B** cytoplasmic (60 µg) fractions were separated on 10% SDS-PAGE gels and analyzed by western blot with anti-Nrf2, anti-HO-1, anti-THOC1, and anti-LDHB. Densities of the Nrf2 and HO-1 bands were evaluated and data expressed as a percentage of CA1 (mean ± SD, *n* = 4–6). *p < 0.05, ** < 0.01, ***p < 0.001. **C**, **D** Representative western blots for Nrf2 and HO-1 together with reference proteins

Nuclear and cytoplasmic fractions (of 30 and 60 µg respectively) were separated by 10% SDS-PAGE, transferred to a nitrocellulose membrane (Amersham), and analyzed by western blotting using the following antibodies: Nrf2 (Proteintech), Keap1 (Proteintech), HO-1 (Cell Signaling Technology), GCLC (Santa Cruz Biotechnology), GCLM (Santa Cruz Biotechnology), and GPx1 (Cell Signalling). Equal protein loading was confirmed by using THOC (Proteintech) and LDHB (Proteintech). Protein bands were detected with horseradish peroxidase-coupled secondary antibodies (rabbit anti-mouse and goat anti-rabbit) and enhanced with a chemiluminescent substrate (Amersham ECL western blotting detection reagents, GE Healthcare). Bands were assessed by densitometry, and normalized with respect to values obtained with the relevant reference protein, except in the case depicted in Fig. 2, in which data were normalized to CA1.

### Organotypic Hippocampal Culture

Organotypic hippocampal cultures were used to examine the effect of N-methyl-D-aspartic acid (NMDA) and pharmacological activation of Nrf2 on neuronal survival in the CA1 hippocampal region. Hippocampal slices were prepared from 6-7-day-old Wistar rats using the slightly modified Stoppini method [43]. Briefly, isolated hippocampi were cut into 400 µm slices using a McIlwain Tissue Chopper and transferred to Millicell-CM (Millipore) membranes for further growth. Slices were cultured in medium containing 50% Neurobasal (Gibco), 24% horse serum (Gibco), 20% HBSS (Gibco), B-27 supplement (Gibco, 1:50), 1 M HEPES (Gibco), 5 mg/mL glucose (Sigma), 0.5 mM glutamax (Gibco), and antibiotic antimycotic solution (Sigma, 1:100). Cultures were started in a medium containing 24% horse serum, which was removed gradually starting from DIV three to seven. Cultures were maintained in a humidified atmosphere of 5% CO_2_ at 36°C for 8 days. Neuronal death was induced using 25 µM NMDA (3 h in culture). The Nrf2 activator sulforaphane at doses of 5 and 10 µM was administered together with NMDA, or 15–30 min after its administration. After 3 h of incubation, NMDA was removed, whereas sulforaphane was present in the culture medium until the end of the experiment. Cell death was performed 24 h after NMDA injury by measuring the intensity of the fluorescent cell-death marker propidium iodide (PI). Values were normalized to the maximal fluorescence intensity obtained by treating slices with 100 µM NMDA.

### Hematoxylin and Eosin Staining

Gerbils from the control, as well as the groups termed ischemic (I/R NaCl), and ischemic subjected to sulforaphane (I/R SFN and I/R 30’SFN), were treated in the above manner. After seven days of recovery, the animals were perfused with 4% paraformaldehyde. Brains isolated from them were then subject to dehydration in graded ethanol and xylene baths, and embedded in paraffin. Sections 5–7 µm thick were stained with hematoxylin and eosin, before morphometric evaluation of neurons within the CA1 region was performed by counting intact neurons from ten well-defined 250-µm fields in this region using Zen 3.0 counting software.

### Statistics

**Fig. 3:**
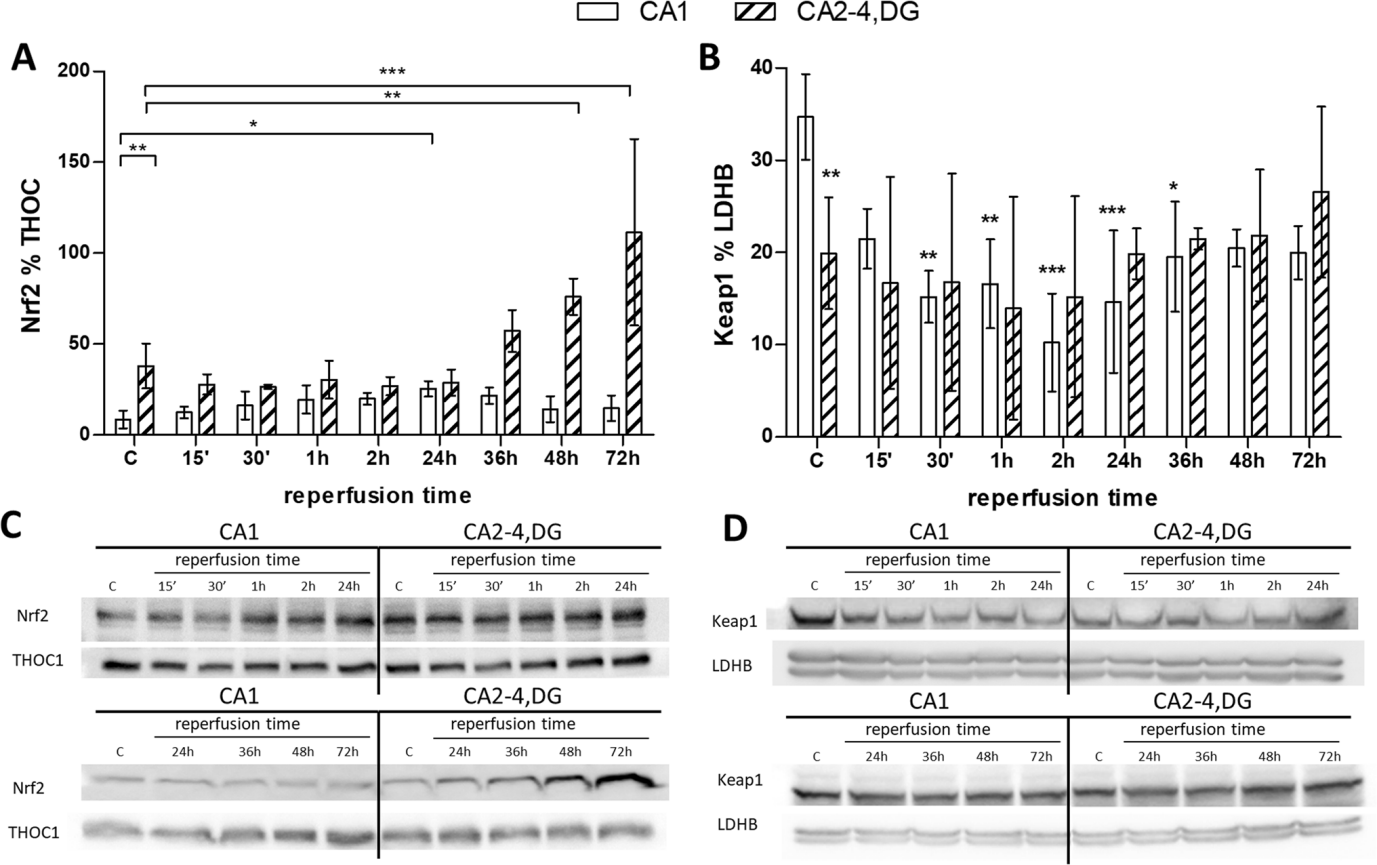
Post-ischemic changes in Nr2 activity and Keap1 immunoreactivity in CA1 and CA2-4,DG (as ischemia-vulnerable and resistant) hippocampal regions of control (C) and I/R animals (15, 30 min or 1, 2, 24, 36, 48, and 72 h following 5 min of ischemia). **A** Nuclear (30 µg) or **B** cytoplasmic (60 µg) fractions were separated on 10% SDS-PAGE gels and analyzed using western blot with anti-Nrf2, anti-Keap1, anti-THOC1, and anti-LDH. Densities of Nrf2 and Keap1 bands were evaluated and data expressed as a percentage of THOC1 and LDHB, respectively (mean ± SD, *n* = 4–5). *p < 0.05, ** *p* < 0.01, ***p < 0.001. **B** Statistical significance vs. control CA1. **C**, **D** Representative western blots for Nrf2 and Keap1 together with reference proteins

**Fig. 4:**
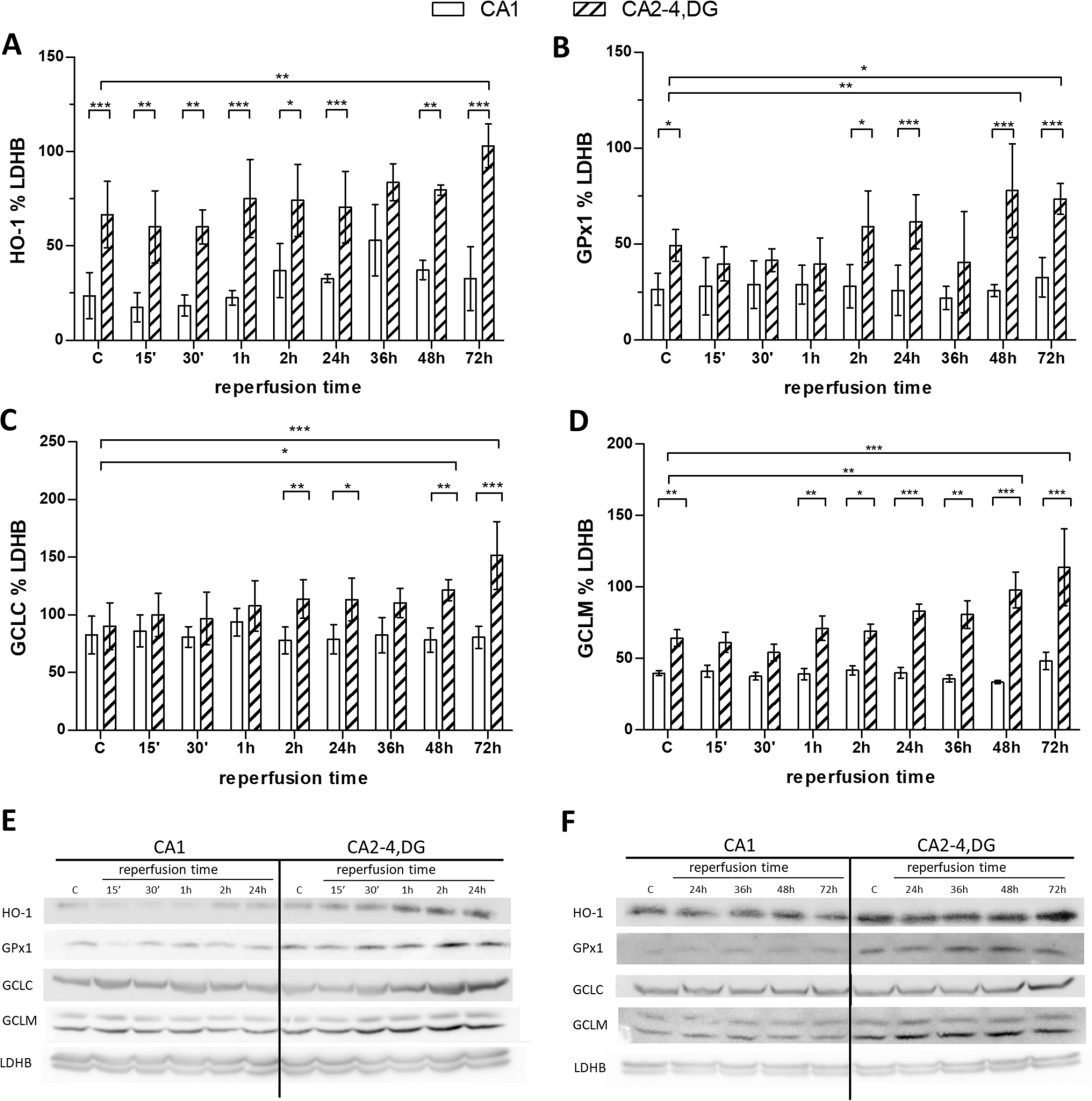
Post-ischemic changes in heme oxygenase 1 (HO-1), glutathione peroxidase 1 (GPx1), glutamate-cysteine ligase catalytic sub-unit (GCLC) and glutamate-cysteine ligase modifier sub-unit (GCLM) immunoreactivity in the CA1 and CA2-4,DG (as ischemia-vulnerable and resistant) hippocampal regions of control (C) and I/R animals (15, 30 min, or 1, 2, 24, 36, 48, and 72 h following 5 min of ischemia). Cytoplasmic fractions (60 µg) were separated on 10% SDS-PAGE gels and analyzed using western blot with anti-HO-1 (**A**), anti-GPx1 (**B**) anti-GCLC (**C**), anti-GCLM (**D**), and anti-LDHB. Densities of bands were evaluated and data expressed as a percentage of LDHB (mean ± SD, *n* = 4–5). *p < 0.05, ** *p* < 0.01, ***p < 0.001. **E**, **F** Representative western blots for HO-1, GPx1, GCLC, GCLM, and together with LDHB as reference protein

**Fig. 5:**
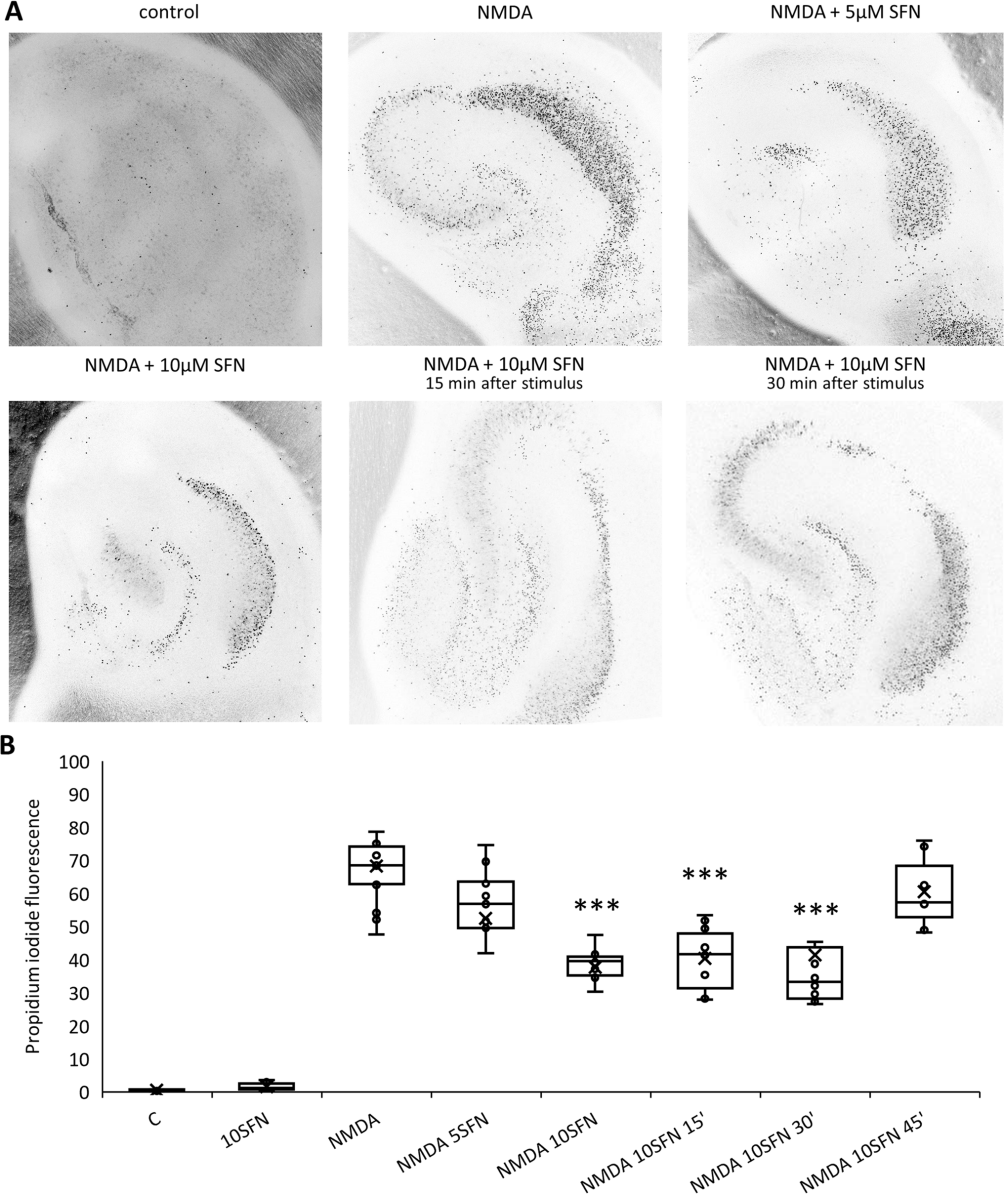
Sulforaphane (SFN), an activator of Nrf2, suppresses neuronal death in CA1 in organotypic hippocampal culture subjected to excitotoxic injury. **A** Color-inverted fluorescent images of propidium iodide-stained hippocampal slices 24 h after treatment with 25 μM NMDA alone or together with 5 or 10 µM SFN. SFN was applied 0, 15, 30, or 45 min after NMDA treatment. **B** Cell death was measured by reference to propidium iodide fluorescent intensity. The results are expressed as the median plus interquartile range. *n* = 7–14, ***p < 0.001

**Fig. 6:**
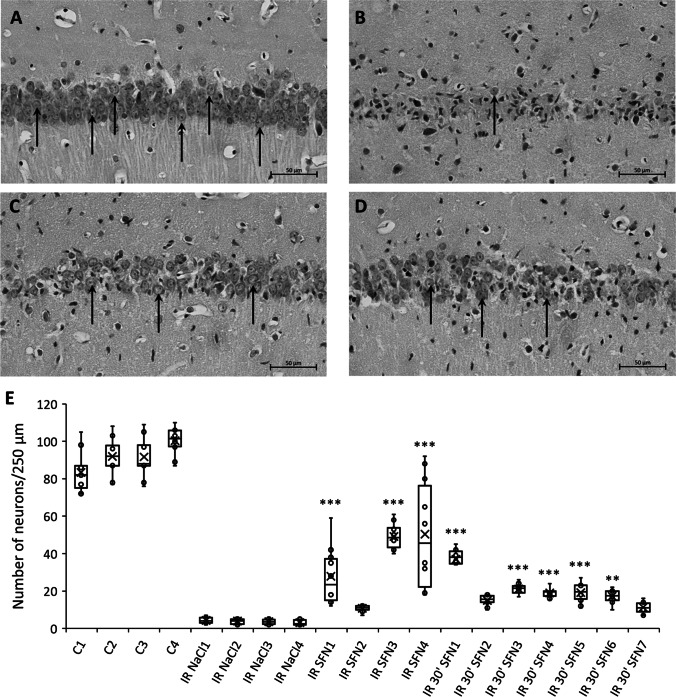
Pharmacological activation of Nrf2 suppresses neuronal cell death in the CA1 region following transient cerebral ischemia in the gerbil model. A single dose of SFN (5 mg/kg) or saline were injected intraperitoneally during the ischemic procedure or 30 min of reperfusion. Seven days after ischemia-reperfusion (I/R) animals were perfused with ice-cold 4% paraformaldehyde. Dissected brains were used for histological examinations on paraffin-embedded sections stained with hematoxylin and eosin. Enlarged area of pyramidal cells in CA1 region in control animals (**A**), ischemic animals treated with saline (I/R NaCl) (**B**), and ischemic animals treated with SFN during ischemic procedure (I/R SFN) (**C**) or 30 min of reperfusion (I/R 30’SFN) (**D**). **E** The extent of cell damage of CA1 hippocampal regions was measured by counting intact neurons from ten well-defined 250-µm fields in this region (some marked by arrows). The results are expressed as the median plus interquartile range. (*n* = 10), p** < 0.01, ***p < 0.001, scale bar: 50 µm

GraphPad Prism 5 software was used to perform statistical analysis. The data are expressed as the mean ± SD (Figs. 2, 3, 4), or the median with interquartile range (Figs. 5 and 6). The significance of differences among groups was calculated using one-way ANOVA and a multiple comparison Bonferroni test. A value of *p* < 0.05 was considered significant.

## Results

### The Nrf2 Pathway Activity Differs Between the CA2-4,DG and CA1 Regions of the Gerbil Hippocampus, Both in The Control and in Animals Sustaining I/R Episode

Western blot analysis of nuclear Nrf2 levels served as a measure of Nrf2 activity in the various brain regions. As Fig. 2A and C demonstrate, Nrf2 levels were significantly higher in the ischemia-resistant part of the hippocampus (CA2-4,DG) than in the ischemia-vulnerable sector (CA1) in the control group. And as with CA2-4,DG, nuclear Nrf2 levels are also higher in the olfactory bulb, while in the cortex, striatum, and cerebellum they are similar to the ones observed in the CA1 region. Furthermore, we examined the immunoreactivity of heme oxygenase 1 (HO-1) as an Nrf2 target protein in the tested regions. As Fig. 2B and D show, HO-1 levels were found to correlate with Nrf2 activation. The only difference was observed in the olfactory bulb, where high Nrf2 activity was accompanied by low HO-1 immunoreactivity (a matter discussed further in what follows). In conclusion, and not in line with expectations, the control was characterized by a higher level of Nrf2 activity in CA2-4,DG than in CA1.

The effects of I/R episode on Nrf2 activity in both hippocampal regions were then studied. As Fig. 3A and C show, I/R had no demonstrable effect on Nrf2 activity in CA2-4,DG until 36 h of reperfusion had passed, while a stimulatory effect on Nrf2 nuclear level was observed after 48 and 72 h of reperfusion. The effect of I/R episode on Nrf2 activity was quite different in CA1. There, an increase in Nrf2 activity (comparable to that in CA2-4,DG) was only observed 24 h after reperfusion, with this returning to the control level in subsequent hours. In conclusion, I/R episode can be said to have affected Nrf2 activity in the hippocampal regions differentially. In CA2-4,DG, long-lasting hyperactivation of Nrf2 is observed at late times of reperfusion, whereas in CA1, Nrf2 activation is temporary and occurs 24 h after I/R episode.

The reasons for these regional differences in Nrf2 activity are unclear, but seem to link up reciprocally with regional differences in cytoplasmic levels of Keap1. As mentioned earlier, that is a cytoplasmic suppressor of Nrf2 that prevents the latter’s translocation to the nucleus [28]. To verify the hypothesis, Keap1 levels were measured using western blot analysis of the cytoplasmic fraction in the two hippocampal regions. As Fig. 3B and D show, the cytoplasmic Keap1 level was significantly lower in CA2-4,DG than in CA1 in the control gerbils. While I/R has no effect on Keap1 levels in CA2-4,DG, it results in a time-dependent decrease in Keap1 immunoreactivity in CA1. This indicates that changes in immunoreactivity are in line with those in Nrf2 activity.

### Correlation Between Nrf2 Activity and Its Target Proteins

To demonstrate whether Nrf2 activation in CA2-4,DG correlates with greater immunoreactivity of its target proteins western blot analyses were performed for heme oxygenase 1 (HO-1), glutathione peroxidase 1 (GPx1), the glutamate-cysteine catalytic sub-unit (GCLC), and the glutamate-cysteine ligase modifier sub-unit (GCLM) (Fig. 4). HO-1 is involved in the production of the essential physiological antioxidant bilirubin [44] and such other products as carbon monoxide and free iron, while GPx1, GCLC, and GCLM engage in the maintenance of glutathione homeostasis [45].

In the case of heme oxygenase (Fig. 4A), immunoreactivity was higher in CA2-4,DG than in CA1, in a difference that was evident from the control group and persisted for up to 72 h of reperfusion. Interestingly, a slight and transient increase in HO-1 was seen 36 h after I/R in the CA1 region, which may be related to the previously described increase in Nrf2 activity in this region 24 h after reperfusion. In CA2-4,DG, a significant increase in HO-1 was observed after 72 h of reperfusion, consistent with a peak in Nrf2 activity in this region.

Differences in immunoreactivity between CA1 and CA2-4,DG were already marked in controls and persisted in all studied groups for GPx1 (Fig. 4B) and GCLM (Fig. 4D). The immunoreactivity of these did not change over time in CA1, even as in the case of CA2-4,DG, there was a gradual increase from 2 h (GPx1) or 1 h (GCLM) following I/R episode, this with reaching a peak at 48–72 h into I/R.

In the case of GCLC, differences between CA1 and CA2-4,DG became apparent 2 h after I/R episode and persisted for up to 72 h (Fig. 4C).

The higher immunoreactivity of target proteins in CA2-4,DG as opposed to in CA1 confirms the higher level of activity of Nrf2 in CA2-4,DG under basal conditions.

### The Neuroprotective Effect of Pharmacological Activation of Nrf2 in In vitro and In vivo Models of Cerebral Ischemia

To verify the effect of pharmacological activation of Nrf2, the commonly-used activator sulforaphane (SFN) was deployed in two well-known models of cerebral ischemia, i.e., in vitro *—*excitotoxicity injury in organotypic hippocampal slices; and in vivo -5-min bilateral carotid-artery occlusion in gerbils.

The exposure of organotypic hippocampal slices to 25 µM NMDA resulted in 70 ± 10% cell death expressed as a percentage of maximum fluorescence, with the CA1 region mainly affected (Fig. 5). SFN applied at a concentration of 5 µM together with NMDA had no effect on neuronal mortality, whereas at a concentration of 10 µM it reduced neuronal damage considerably to approximately 40 ± 10% (Fig. 5B). A similar protective effect was observed when SFN (10 µM) was administered for 15 or 30 min after NMDA administration. However, administration of SFN (10 µM) 45 min after NMDA offered no apparent protective benefits. SFN (10 µM) applied alone proved inert in its effects on hippocampal slices.

Where the gerbil model of transient cerebral ischemia was concerned, control animals 7 days on from the sham operation had pyramidal cells in CA1 exhibiting proper morphology, even as morphometric measurements revealed mean numbers of normal neurons equal to 92 ± 7.5 per 250 μm (mean ± SD, *n* = 4) (Fig. 6A, E). In the ischemia-operated groups, only 4 ± 0.6 (mean ± SD, *n* = 4) neurons remained intact (Fig. 6B, E). In groups treated with 5 mg/kg sulforaphane, the morphology of the *stratum pyramidale* of the CA1 region was significantly improved in comparison to ischemia-operated group, with mean numbers of proper cells being 35 ± 19 (± SD, *n* = 4) and 20 ± 7 (± SD, *n* = 7), respectively, for subjects injected during ischemia (Fig. 6C, E) or 30 min into reperfusion (Fig. 6D, E). The morphology of the CA2-4,DG region did not reveal change between the ischemia-operated, SFN-injected and control groups (data not shown).

In conclusion, pharmacological activation of Nrf2 can be seen to have protected hippocampal neurons within the CA1 region from I/R episode, in both in vitro and in vivo models.

## Discussion

It may be of scientific and translational significance to explain the differential vulnerability of the hippocampal regions to I/R episode. In the gerbil model used in this study, the hippocampal CA1 proves particularly vulnerable, while CA2-4,DG demonstrates notable resistance to I/R [7, 12, 46]. Previous studies investigating the mechanism underpinning such results have addressed regional differences in glutamate neurotoxicity, calcium-signalling, the expression of various genes, mitochondrial function, and ROS production; but they have yet to clarify all relevant issues [8, 12, 42, 46, 47]. The study presented here concentrated on endogenous neuroprotection as a mechanism leading to ischemia-resistance of CA2-4,DG, with the focus being on the role in this phenomenon attributable to the transcription factor Nrf2. The two main aims of the research have been to understand how the brain protects itself, and to seek to draw on this knowledge to induce endogenous neuroprotection in stroke treatment.

### The Role of Nrf2 in Endogenous Neuroprotection

Regulation of endogenous neuroprotection is based, not on a single mechanism, but on the concerted action of multiple cellular and molecular pathways that counteract the mechanisms induced during I/R episode. These include activation of antioxidant and anti-inflammatory strategies, adaptation of energy metabolism, and enhancement of neuroprotective and/or regenerative mechanisms [48]. The simultaneous activation of these processes due to I/R episode results in a shift from cell death to cell survival mode [49]. And one factor that acts as a master cellular regulator and could potentially serve as such a switch is the transcription factor Nrf2. Here, we show that CA2-4,DG has higher Nrf2 activity than CA1 even in the circumstances of control animals, with this difference persisting after I/R episode (Figs. 2A, 3A). High Nrf2 activity is reflected in the higher immunoreactivity of Nrf2 target proteins such as HO-1, GCLC, GCLM, and GPx1, in CA2-4,DG as compared with CA1 (Fig. 4). This confirms, on the one hand, that the high nuclear Nrf2 content in CA2-4,DG translates into its activity in this region; and on the other that antioxidant processes dominate in CA2-4,DG. Indeed, the experiments detailed here have been in a position to show how immunoreactivity of GPx1, GCLC, and GCLM dominates in CA2-4,DG as compared with CA1, whether in control-animal circumstances or following on from I/R episode (Fig. 4B–D). We may therefore be dealing with a major component of the protection against oxidative stress that Nrf2 is able to supply. The three aforementioned enzymes are involved in the synthesis and maintenance of glutathione. GCLC and GCLM together catalyze the rate-limiting step in glutathione biosynthesis [45], while GPx1 — as a detoxifying enzyme — produces oxidized glutathione. Unsurprisingly, glutathione levels are lower in cells in which Nrf2 is disrupted, while activation of Nrf2 by genetic or pharmacological factors leads to increased glutathione levels [50]. Although we did not measure glutathione levels or other markers of oxidative stress, relevant data on differences within the hippocampal regions indicate, on the one hand, a dominance of oxidative stress in CA1 compared to CA3 [51, 52] and, on the other, a greater efficiency of the antioxidant system in CA3 than in CA1 [53], and hence a result in line with our own.

Where GCLC, GLCM, and GPx1 were concerned, dominance of immunoreactivity in CA2-4,DG as compared with CA1 was also observed for HO-1 (Fig. 4A). This cytoprotective enzyme catalyzes the rate-limiting step in heme degradation, leading to the generation of the biliverdin, carbon monoxide and free iron known to regulate important biological processes. Biliverdin is rapidly reduced to bilirubin, a potent antioxidant; carbon monoxide regulates inflammation, apoptosis, cell proliferation, fibrosis and angiogenesis; while iron induces the synthesis of ferritin — as a protective enzyme that sequesters iron ions [44]. Several studies have shown that an increase in HO-1 expression due to Nrf2 activation is an important component of Nrf2-induced neuroprotection [54]. In this context, the low HO-1 immunoreactivity despite high Nrf2 activity we observed in the olfactory bulb may prove surprising (Fig. 2B). However, the likely reason by which to account for the phenomenon is the high level of the transcriptional regulatory protein Bach1 in the olfactory bulb, given the capacity to inhibit HO-1 transcription [55].

In conclusion, the results presented here support the idea of Nrf2 being involved in the endogenous neuroprotection of CA2-4,DG.

### The Role of Nrf2 in Cerebral Ischemia

A further topic addressed in this work detailed here involved changes in Nrf2 activity following on from I/R episode. We were able to demonstrate a differential effect of I/R on Nrf2 levels in the two different hippocampal regions (Fig. 3A). While in CA2-4,DG, hyperactivation of Nrf2 was observed 48 h after I/R, and persisted for up to 72 h, in CA1 there was short-term activation of Nrf2 24 h after reperfusion, only for this to return to control levels in the following 24 h. Based on Liu’s review, it seems that most studies on focal ischemia models are consistent with our results, confirming Nrf2 pathway activation after ischemic injury [27]. For example, in the penumbra, a parallel Keap1 decrease and Nrf2 increase in protein expression were noted 2 h after 60 min of middle cerebral-artery occlusion (MCAO) in mice [56]. Moreover, increased expression of antioxidant proteins, such as thioredoxin, glutathione and HO-1 was shown 24–72 h after MCAO. Meanwhile, a similar trend for changes in the expression of Keap1, Nrf2, and antioxidant proteins was observed in the ischemic core, even if the responses involved were much less intense [56]. In contrast, 24 h after 60 min of MCAO in rats, Nrf2 upregulation had occurred in the penumbra, but not in the core of ischemia [57]. In addition, increased expression of Nrf2 and its target gene HO-1 was observed in rats 24 h after MCAO for 70 min, only to be followed by a decrease below control levels 72 h on from I/R [71].

In contrast, analysis of global ischemia studies provides a heterogeneous picture in terms of the dynamics of change in Nrf2 activity after ischemia. These discrepancies may reflect differences in experimental protocols, including as regards type of animal, duration of ischemia, the presence or absence of reperfusion, follow-up time after ischemia, and site of sample. The only other study using a gerbil model showed an increase in Nrf2 and HO-1 in CA1 after 48 h of reperfusion [58]. However, as that was in fact the only time point studied in the experiment dynamics characterising changes in this case many not be discussed. In C57BL6 mice subjected to bilateral common carotid-artery occlusion for 20 min, and 24 h of reperfusion, an increase in Nrf2 expression in the striatum was accompanied by increases in levels of HO-1, GCLC, GCLM, and NAD(P)H quinone dehydrogenase (NQO1) [59]. In contrast, 3 days after two vessel occlusions (of 20 min) total, cytoplasmic and nuclear Nrf2 protein levels were elevated in rat hippocampal CA1, although this increase failed to achieve statistical significance [60]. The study also failed to observe any increase in expression of HO-1. Similarly, a slight increase in cytoplasmic (though not nuclear) levels of Nrf2 in hippocampal CA1 was observed between 6 and 72 h on from 15 min of four-vessel occlusion in rats, albeit with no significant changes noted for Nrf2-reagulated proteins including HO-1, NQO1, superoxide dismutase (SOD2), and GPx1 [61].

In conclusion, the current state of knowledge does not offer a clear indication as to the functional significance in cerebral ischemia of changes involving Nrf2. However, the dynamics we note for its activity following I/R episode are consistent with our hypothesis that Nrf2 is involved in the endogenous neuroprotection of CA2-4,DG. Its activation in this region is present even under control conditions, with hyperactivation noted 48 h after I/R. Moreover, the dynamics to changes in Nrf2 activity in this region resemble the biphasic profile of protection described in ischemic preconditioning [48, 62], whereby early protection is mobilized within minutes of injury even as late protection ensues 24–48 h after I/R [6, 49]. In ischemic preconditioning, early (also known as rapid or classical) protection is short-lived, and is based mainly around changes in activity and post-translational modifications of existing proteins, while late (also known as delayed or protein-synthesis-dependent) protection is more durable in nature and involves de novo protein synthesis. By reference to this analogy, we speculate that the control of Nrf2 activity observed in CA2-4,DG affords this region early protection, while the subsequent Nrf2 hyperactivation occurring 48 h after I/R episode provides late protection.

Equally, with respect to CA1, it can be assumed that the changes in Nrf2 activity we observed (Fig. 3A) may reflect transient activation of neuroprotective mechanisms. The gradual activation of Nrf2 that can be noted, peaking after 24 h of reperfusion, did not translate into an increased level of the target proteins chosen for study. And this activation was most likely too small to offer effective protection of the CA1 region against I/R damage. Another possible explanation would be long-lasting inhibition of the protein synthesis pathway of the kind demonstrated previously through analysis of metabolic differences between CA1 and CA2-4,DG [12]. As we know from data shown in Figs. 5 and 6, it was only the pharmacological activation of Nrf2 that protected hippocampal CA1 neurons effectively after I/R, even when administered late in relation to the ischemic episode.

### “Physiological” Activation of Nrf2

The “physiological” activation of Nrf2 in our model is also worth examining. In this regard, a view held widely has cellular activation of the Nrf2 system occurring in response to a stress factor. Under oxidative stress, Keap1 is modified through an oxidative-dependent mechanism, and releases Nrf2, which translocates into the nucleus, where it activates the expression of many genes. Surprisingly, our results showed that, under control conditions, the Nrf2 pathway was activated in CA2-4,DG as opposed to in CA1. This correlates with Keap1 levels that are lower in ischemia-resistant regions than in CA1, albeit thanks to a mechanism that is not known. The constitutive activation of Nrf2 has been described in many human cancers, while hyperactivation of the factor is shown to promote cell proliferation and metabolic reprogramming, as well as conferring cellular resistance to cancer therapies [63].

### Nrf2 as a Promising Therapeutic Target Against Cerebral Ischemia

Our work as described here shows a protective effect arising out of pharmacological activation of Nrf2 in the hippocampal CA1 region following I/R episode (Figs. 5 and 6). We found a dose-dependent response to the administration of sulforaphane when it came to on neuronal viability in in vitro studies, with an optimal effect noted where the dose was 10 µM. Moreover, a protective effect was evident even when sulforaphane was administered with delay to ischemia in in vivo study (Fig. 6). This is in line with a previous studies demonstrating with various models of cerebral ischemia that pharmacological activation of Nrf2 exerts protective effects. This includes sulforaphane, which will be discussed below, as well as other Nrf2 activators, including dimethyl fumarate [64, 65], tert-butylhydroquinone [66, 67], metformin [68, 69], resveratrol [70, 71], and many others [72].

In in vitro studies, for example, the administering of sulforaphane has been shown to activate Nrf2 signaling and increase the cellular viability of cortical and hippocampal neurons, as well as astrocytes exposed to oxygen–glucose deprivation [73–75].

Analogous outcomes have also been claimed for in vivo models. In the rat MCAO model, pre-treatment with sulforaphane (5 mg/kg, injected intraperitoneally) increased expression of Nrf2 and HO-1, and prevented BBB disruption, lesion progression and behavioral impairment [35]. In turn, a single intraperitoneal administration of sulforaphane (of 5 mg/kg) 15 min after the onset of ischemia was shown to achieve a significant reduction in infarct volume in the context of the rat common carotid/middle cerebral-artery occlusion model [15]. A protective effect was also observed in a rat model of neonatal hypoxia and ischemia in which sulforaphane pre-treatment (5 mg/kg, intraperitoneal injection) reduced caspase-3 and oxidative factors, including levels of malondialdehyde and 8-hydroxy-2-deoxyguanosine [18]. In experimental piglets too, the administration of sulforaphane (10 mg/kg injected intravenously) 15 min after hypoxia–ischemia-induced episode evoked neuroprotection in the highly sensitive part of the putamen and sensorimotor cortex [36]. Interestingly, Nrf2-deficient mice subjected to intracerebral hemorrhage (ICH) demonstrated neurologic deficits after ICH, and were not shown to benefit from the protective effect of sulforaphane [34].

As can be seen from the above examples, the targeting of Nrf2 has become an attractive therapeutic strategy in the treatment of cerebral ischemia [76]. It is worth noting that, currently, a stable synthetic form of sulforaphane, SFX-01, is being validated in clinical trial for the treatment of subarachnoid haemorrhage [77].

## Conclusion

In conclusion, we suggest that high levels of nuclear Nrf2 activity in CA2-4,DG may guarantee resistance of this region to I/R episode, while at the same time offering a potential explanation for the phenomenon of the differential sensitivities of hippocampal regions.

Furthermore, our results are in line with the existing view that Nrf2 activation may represent a promising therapeutic strategy against cerebral ischemia. The uniqueness of Nrf2 lies in its pleiotropic action and subsequent regulation of multiple cytoprotective pathways. This may support more efficient neuroprotection compared to single-target strategies. However, further research is needed before success in treating cerebral ischemia can be declared.

## Data Availability

The datasets generated during and/or analyzed during the current study are available from the corresponding author on reasonable request.
